# Immunocytokines in cancer immunotherapy: opportunities and challenges

**DOI:** 10.3389/fonc.2025.1716612

**Published:** 2026-01-06

**Authors:** Shubo Wang, Lisha Yao, Jianying Cai, Huiquan Gao, Fuxin Zhou

**Affiliations:** 1 Department of Gynecology, The Affiliated Weihai Second Municipal Hospital of Qingdao University, Weihai, China; 2 Department of Thoracic Surgery, The 970th Hospital of the People’s Liberation Army (PLA), Weihai, Shandong, China; 3 Department of Radiotherapy, The Affiliated Yantai Yuhuangding Hospital of Qingdao University, Yantai, China; 4 Department of General and Visceral Surgery, Faculty of Medicine, Medical Center – University of Freiburg, Freiburg, Germany

**Keywords:** cancer immunotherapy, cytokines, immunocytokines, cytokine engineering, antibodies

## Abstract

The clinical use of cytokines is restricted by dose-limiting toxicities (DLT), short half-life, and rapid renal clearance, which collectively hinder administration at therapeutically effective doses. Cytokines fused to antibodies (antibody–cytokine fusion proteins, or immunocytokines) have emerged as a promising strategy to overcome these limitations by directing cytokine payloads to the tumor microenvironment, thereby enhancing antitumor immune responses while reducing off-target effects. Various antibody formats, including intact IgGs and IgG fragments, have been engineered to target tumor-associated cell-surface antigens or extracellular matrix (ECM) components, and fused to a wide range of cytokines. Preclinical studies consistently demonstrate enhanced antitumor efficacy and reduced systemic toxicity compared to unconjugated cytokines, with further synergistic effects observed when combined with chemotherapy, radiotherapy, or immune checkpoint inhibitors. Recent advances include anti-PD-1–based immunocytokines that selectively deliver cytokines to intratumoral CD8^+^ T cells, restoring their function and driving potent antitumor activity. Despite encouraging results, efficacy and safety concerns remain significant challenges for clinical application. Strategies such as cytokine engineering, prodrug approaches, and rational molecular design are being pursued to enhance therapeutic outcomes while minimizing side effects. This review summarizes the conceptual framework, structural design principles, preclinical and clinical progress, current limitations and potential strategies for future development of immunocytokines in cancer immunotherapy.

## 1 Introduction

The idea of exploiting the immune system to eradicate neoplastic cells can be traced back to the nineteenth century (1). Harnessing the immune system for cancer therapy has revolutionized the field of oncology and increasingly gained attention since the twentieth century, paving the way for modern immunotherapeutic strategies. The immune system has the capacity to recognize and destroy malignant cells, and its activity is regulated by multiple molecular signaling pathways. Among these, cytokines play a fundamental role as intercellular messengers that orchestrate leukocyte activation, proliferation, differentiation, effector functions, and survival (2).

Owing to their essential functions in immune regulation, cytokines have long been investigated and applied as therapeutic agents in oncology. The first cytokine therapy, interleukin-2 (IL-2, aldesleukin), was approved by the U.S. FDA in 1992 for the treatment of metastatic renal cellcarcinoma and later in 1998 for metastatic melanoma (3). Rosenberg and colleagues administered IL-2 intravenously at high doses (720,000 IU/kg every 8 h for up to 14 doses over 5 days), achieving an overall objective response rate (ORR) of 14%, including 5% complete responses and 9% partial responses in patients with metastatic renal cell carcinoma. Notably, some responses were durable, with complete remissions (CRs) persisting for many months after therapy. However, high-dose IL-2 therapy can cause severe, life-threatening toxicities, such as hypotension and vascular leak syndrome (VLS). In addition, interferon-*α* (IFN-*α*) was another cytokine approved for the treatment of human cancers, first for hairy cell leukemia (HCL) in 1986, and later for chronic myelogenous leukemia (CML), follicular non-Hodgkin lymphoma (4), melanoma (5) and AIDS-related Kaposi’s sarcoma (6). However, similar to IL-2, the therapeutic application of IFN-*α* was limited by dose-dependent toxicity. At the high doses used in clinical practice, the most common adverse effects included flu-like symptoms such as fever, fatigue, and myalgias, while prolonged administration frequently caused neuropsychiatric complications, including depression and cognitive impairment. Additional toxicities such as hematologic abnormalities, hepatotoxicity, and autoimmune manifestations further limited its clinical use (7, 8).

**Figure 1:**
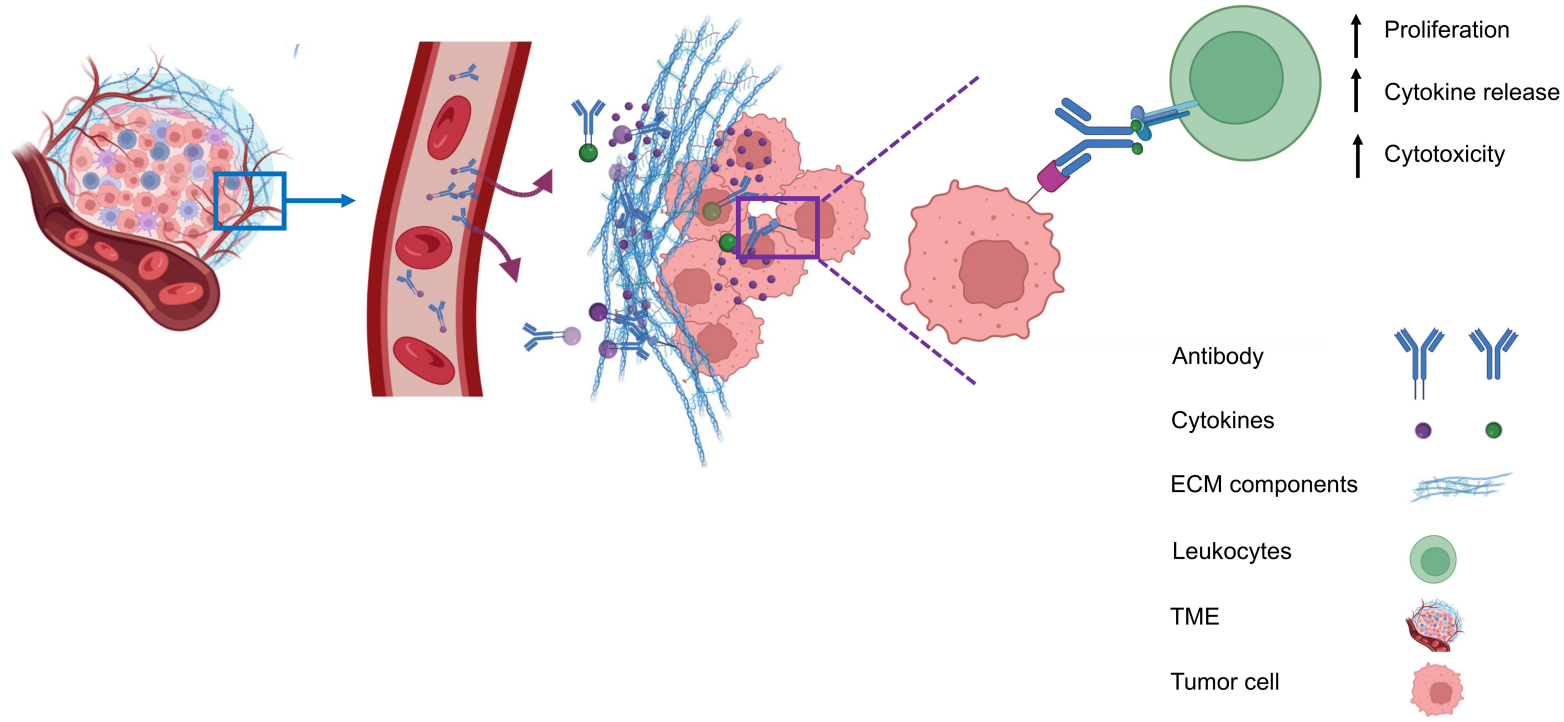
Antibody-mediated delivery of immunocytokines to the tumor microenvironment. Antibodies direct cytokines to tumor-associated antigens, promoting immune cell proliferation, cytokine and release, and cytotoxicity, while reducing systemiv toxicity.

Due to these systemic toxicities, subsequent efforts focused on strategies to confine cytokine activity to the tumor microenvironment. Approaches such as intratumoral or peritumoral cytokine delivery, implantation of cytokine-producing cells, or cytokine gene transfection of tumor cells prior to transplantation were conceived to retain cytokines locally. These strategies produced robust antitumor effects while limiting severe side effects (9–12).Building on the concept of local cytokine retention, antibody-based strategies have been developed to target antigens expressed in the tumor microenvironment (TME) but absent from normal tissues. The antibodies can be exploited to deliver immunostimulatory payloads (such as cytokines) to the tumor microenvironment, enhancing immune cell proliferation, cytokine release, and cytotoxicity, while minimizing systemic toxicity (**Figure 1**).

In mouse models, immunocytokines incorporating immunostimulatory payloads have demonstrated promising activity and selectivity against several tumors. For example, ch14.18–IL2 was able to eradicate metastatic melanoma and neuroblastoma in immunocompetent mice, while also inducing durable, tumor-specific immunity (13). Encouraged by preclinical findings, Hu14.18–IL2 was the earliest immunocytokine to advance into human clinical trials (14). While these studies demonstrated the feasibility of antibody–cytokine fusion proteins, several factors including antibody format, fusion site, and payload selectivity can influence the local concentration of cytokines and the efficacy of immune responses at the tumor site. This review will therefore focus on the different antibody formats and molecular targets, as well as the preclinical and clinical results of immunocytokines and their limitations and potential strategies for future development in cancer therapy.

## 2 Antibody formats and molecular targets

Antibody formats influence the pharmacokinetics, biodistribution, and therapeutic efficacy of immunocytokines. Depending on their structure and the site of cytokine fusion, different formats can influence cytokine stability, tumor penetration, and immune activation. In the following, the major antibody formats used for immunocytokine development are outlined (15).

### 2.1 Antibody moiety formats

**Figure 2:**
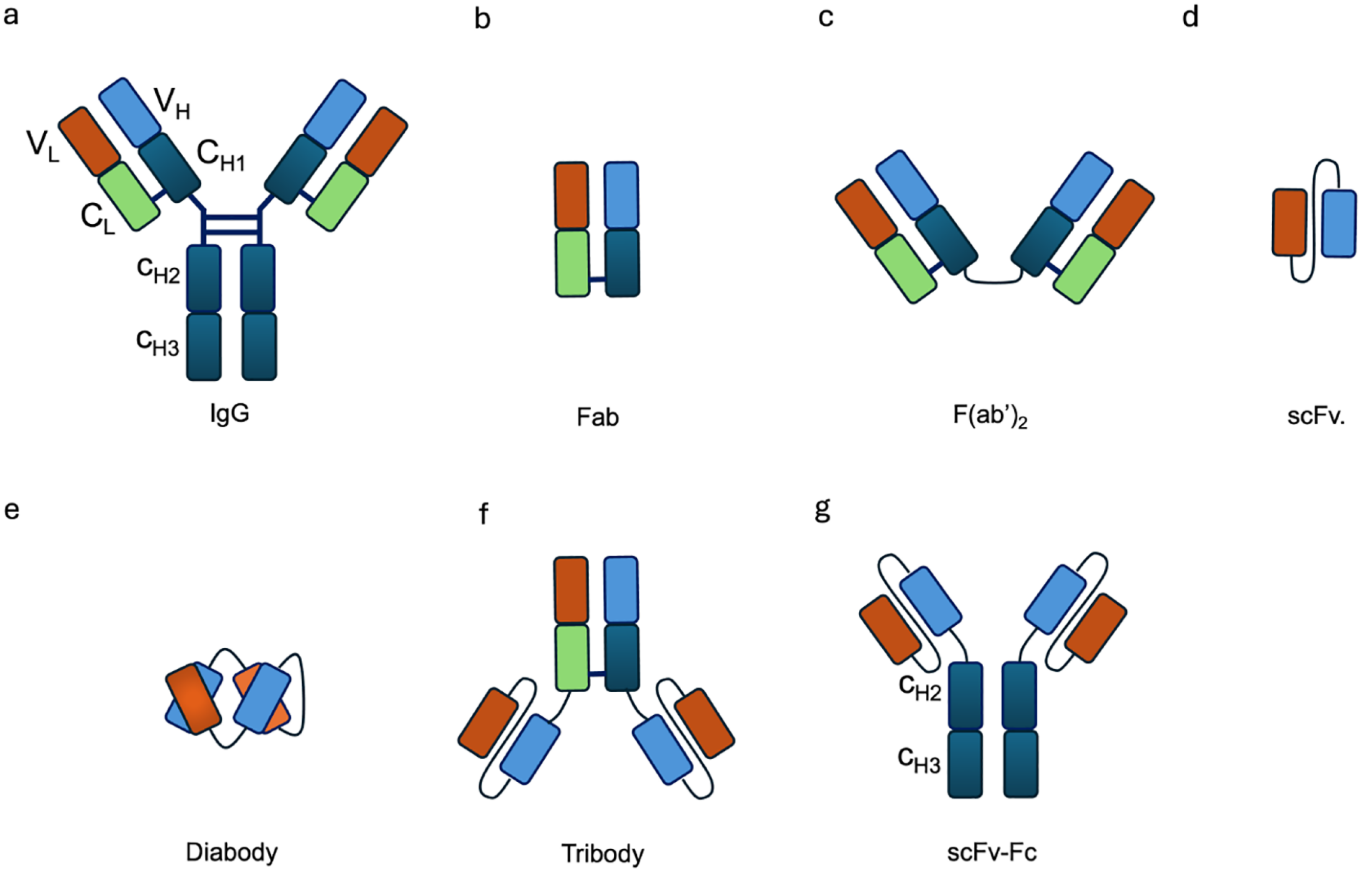
Schematic representation of different antibody formats used in immunocytokine design. **(a)** IgG, **(b)** Fab, **(c)** F(ab)2, **(d)** scFv, **(e)** diabody, **(f)** tribody, and **(g)** scFv-Fc. These formats vary in size, valency, and functional properties, influencing their half-life, tissue penetration, and effector functions.

Generally, antibody moieties in antibody–cytokine fusion proteins can be divided into two categories depending on the involvement of the Fc region. One category comprises large fusion proteins, in which cytokines are fused to the heavy chain of full-length immunoglobulins (IgG, 150 kDa), while the other includes smaller fusion proteins based on antibody fragments (Fab, F(*ab’*)_2_, the single-chain variable fragment (scFv), diabody, tribody, scFv-Fc) (**Figure 2**). The selection of an antibody moiety depends on the relative strengths and limitations of the different antibody formats. Full-length IgG-based immunocytokines provide bivalent binding, long serum half-life through FcRn recycling, and high avidity for their targets, which can enhance tumor retention ()?. In contrast, smaller formats such as scFv, diabody, and Fab fragments exhibit faster tumor penetration within solid tumors, though at the cost of shorter systemic half-life and reduced stability. IgG based immunocytokines benefit from the dual variable regions of intact IgG, which confer high avidity for their specific targets and thereby prolong retention at the tumor site ()?. Moreover, their large molecular size reduces renal clearance, resulting in an extended serum half-life of approximately 21 days ()? (16).

Additionally, the Fc region in an IgG based immunocytokine adds approximately 50 kDa to its size. Fc engagement with the neonatal FcRn triggers IgG recycling and prevents degradation in endothelial lysosomes (17). Moreover, the engagement with Fc*γ* receptors (Fc*γ*Rs) enhances immune responses, eliciting antibody-dependent cellular cytotoxicity (ADCC) or antibody-dependent cellular phagocytosis (ADCP). In ADCC, Fc*γ*R engagement on natural killer (NK) cells activates the release of perforin and granzymes, leading to tumor cell lysis. Fc*γ*R binding on macrophages promotes phagocytosis of antibody-coated targets and contributes to antigen presentation (18). In the context of immunocytokines, cytokine fusion can modulate these Fc-mediated effector functions by altering Fc receptor accessibility, antibody orientation, and local immune activation. For instance, cytokine domains fused to the antibody can enhanceeffector cell recruitment and activation at the target site, thereby indirectly promoting Fc-mediated mechanisms such as ADCC and ADCP, while generally maintaining FcRn mediated recycling and prolonged serum half-life.

In contrast to its pro-inflammatory functions, Fc engagement can also mediate immune suppression through Fc*γ*RIIb, an inhibitory receptor containing an immunoreceptor tyrosine based inhibitory motif (ITIM). Fc*γ*RIIb is broadly expressed on immune cells, where it dampens immune responses. Fc*γ*RIIb also exists in a soluble form that can bind circulating immune complexes, potentially competing with membrane-bound receptors and modulating antigen presentation (19–21). Furthermore, the complement system can be activated through Fc binding to C1q, leading to complement-dependent cytotoxicity (CDC) and the generation of pro-inflammatory mediators (22–24). While the mechanism enhances antitumor immunity, uncontrolled complement activation may also contribute to off-target tissue damage.

However, in addition to the risk of uncontrolled inflammation mediated by Fc functions, the large size of IgG also hinders the distribution and penetration of immunocytokines into tumor tissue (25). To improve extravasation and penetration, the scFv format was developed, consisting of the variable domains of both heavy and light chains (**Figure 2d**). Nevertheless, scFv based immunocytokines undergo rapid renal clearance due to their small molecular size (25–30 kDa), which allows efficient filtration through the glomerulus. As a result, they exhibit a short serum half-life compared to IgG based formats (26). In addition, the monovalent nature of scFvs confers reduced binding avidity toward their targets, leading to faster dissociation rates and less effective tumor retention ()??.

To improve binding avidity, tumor penetration, stability, and pharmacokinetic properties, several antibody fragment formats have been developed, including F(ab)_2_, diabody, tribody, and scFv–Fc (27). A diabody (**Figure 2e**) is a dimeric variant of the scFv, while the tribody (**Figure 2f**) combines the diabody format with an additional F(ab)_2_. The scFv–Fc format was designed to enhance not only binding avidity but also the ability to induce ADCC and ADCP. In spite of these advantages, tumor penetration is reduced due to the larger molecular size (106 kDa) and it brings an increased risk of off-target toxicity when the target antigen is also expressed in normal tissues (28).

### 2.2 Molecular targets

Ideal target antigens for immunocytokines are those abundantly expressed in tumor tissue but minimally or not expressed in normal tissues. Current molecular targets encompass both cellular and tumor microenvironment (TME) components. Several cellular antigens have been investigated for antibody-based immunocytokines, including integrins (*α*v*β*3), prostate-specific membrane antigen (PSMA), vascular endothelial growth factor receptors (VEGFRs), and endoglin (CD105) (29). Among these cellular targets, PD-1, PD-L1, and carcinoembryonic antigen (CEA) have been explored as promising targets for colorectal cancer (30–32). In addition, disialoganglioside 2 (GD2) and fibroblast activation protein (FAP) have been investigated as targets for immunocytokines in several tumor types (33, 34). Immunocytokine programs addressing these cellular targets are at various stages of development, ranging from preclinical studies to ongoing clinical trials.

## 3 Preclinical development of immunocytokines

A number of immunocytokines have been investigated in preclinical mouse models of cancer. The following tables summarize the molecular arrangements of antibody moieties and cytokine payloads, as well as the tumor models. Among these, immunocytokines incorporating IL-2, IL-12, or TNF have demonstrated the most favorable tumor-homing properties and antitumor activity. Encouraged by these promising results, some candidates have progressed to clinical trials (13, 35–37).

### 3.1 IL-2-based immunocytokines under preclinical research

IL-2–based immunocytokines are the most extensively developed, due to their strong capacity to activate the immune system. IL-2 is a small cytokine secreted primarily by activated T cells, promoting the proliferation and differentiation of lymphocytes (38). Based on the clinical evidence of unconjugated IL-2, targeted formats were developed to increase its therapeutic efficacy and safety. Some research on fusion proteins of antibody and IL-2 as a payload demonstrated strong anti-cancer activity in various mouse models of cancer, including metastatic melanoma, neuroblastoma, prostate carcinoma, colon adenocarcinoma, non-small cell lung carcinoma, and lymphoma.

Early studies explored wild-type (WT) IL-2 as a payload for immunocytokines. In melanoma-bearing mouse models, fusion of IL-2 with an antibody specific for GD2 inhibited lung and liver metastases and prolonged survival following lymphokine-activated killer (LAK) cell reconstitution in immunocompromised mice, compared to unconjugated IL-2 administration (13). In neuroblastoma models, anti-GD2 WT IL-2 immunocytokines enhanced antitumor efficacy and immune cell infiltration when delivered intratumorally, compared with intravenous injection or unconjugated IL-2 treatment (39). In addition, Kujawski et al. developed a fully bioactive homodimeric immunocytokine, WT IL-2 fused to the anti-CEA at C-terminus, which is the clinically tested humanized anti-CEA antibody (hT84.66-M5A). In a mouse breast carcinoma model, it showed enhanced tumor inhibition and improved immunity against tumor rechallenge when combined with stereotactic tumor irradiation (40).

However, because of the potential side effects associated with the Fc region of full-length antibodies and the limited tumor penetration of large IgG molecules, researchers have extended immunocytokine development to include smaller antibody fragments, such as scFv formats linked to WT IL-2 and directed against various tumor-associated antigens (TAAs) (9). L19–IL-2 is another widely studied WT IL-2 immunocytokine, consisting of a non-covalent homodimeric scFv format with improved tumor-homing properties and penetration into the TME. It targets the extradomain-B (EDB) of fibronectin, which is primarily expressed during embryonic development and in tumor vasculature (41). In mouse models of orthotopic pancreatic cancer, L19–IL-2 inhibited tumor growth and metastasis and achieved long-term tumor control, with immune-mediated mechanisms supported by increased macrophage and NK cell infiltration in tumor tissue (42).

IL-2 receptors exist in different forms depending on their subunit composition. Regulatory T cells (Tregs) express the full trimeric receptors (*α*, *β*, and *γ* chains), which have much higher affinity than the intermediate *βγ* receptor expressed on NK cells and naïve CD8^+^ T cells. Engineering IL-2 variants with altered affinities for these receptor forms provides greater flexibility in the design of immunocytokines. One such example is a super mutant IL-2 (sumIL-2) with decreased binding to IL-2R*α* and increased binding to IL-2R*β*, which was fused to antibodies targeting the epidermal growth factor receptor (EGFR) or human epidermal growth factor receptor 2 (Her2). In murine tumor models, Erb–sumIL-2 significantlypromoted tumor eradication and induced protective memory immunity (43).

In addition to IL-2 mutants engineered for increased receptor affinity, variants with reduced affinity can also enhance immune responses. Ren and colleagues developed an immunocytokine (PD-1laIL-2) consisting of an anti–PD-1 antibody fused to a mutant IL-2 with low affinity for the IL-2R*α*/*β* complex. This construct exhibited effective tumor inhibition with lower systemic toxicity compared to treatment with anti–PD-1 or IL-2 alone, or their combination (44). Owing to itsreduced affinity for IL-2R*α* and IL-2R*β*, PD-1laIL-2 preferentially expanded intratumoral PD-1^+^CD8^+^ T cells while sparing Tregs, CD4^+^ T cells, and peripheral CD8^+^ T cells (**Table 1**).

**Table 1:** Overview of IL−2–based immunocytokines.

Name	Structure	Target	Tumor models
Anti-CEA-IL2	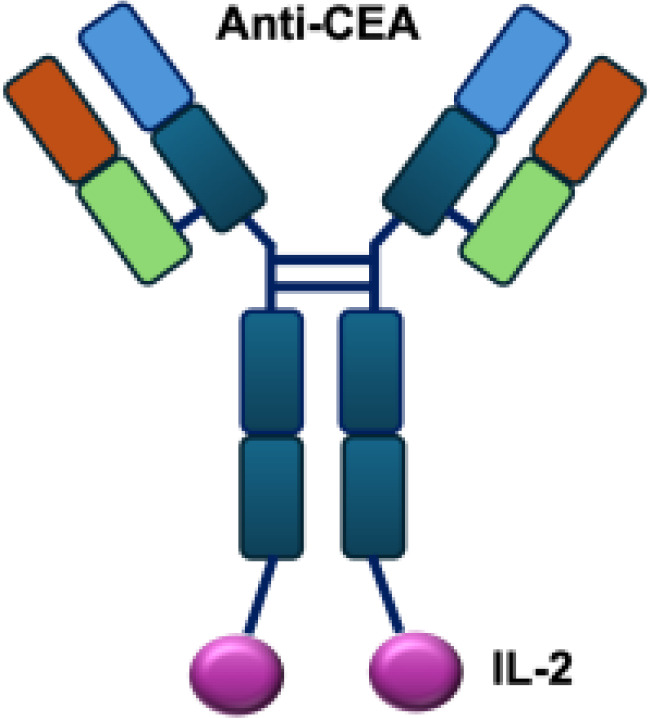	CEA	E0771/CEA; MC38/CEA
CBD-IL2	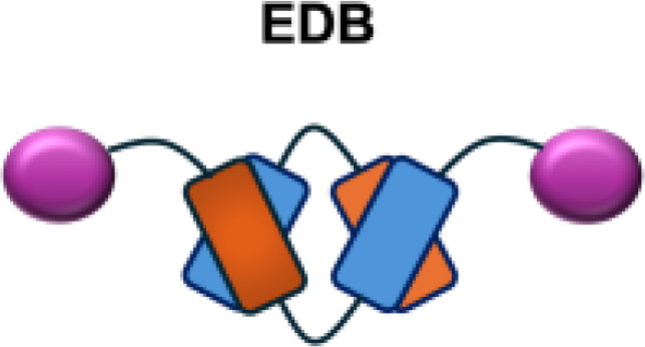	Collagen	B16F10 melanoma; CT26 colon carcinoma; MMTV-PyMT breast cancer
PD-1-laIL2	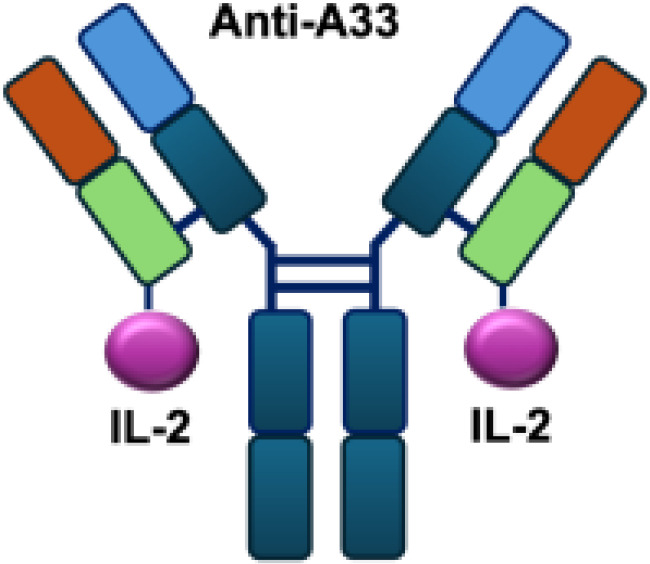	PD-1	MC38; B16F10
Erb-sumIL2	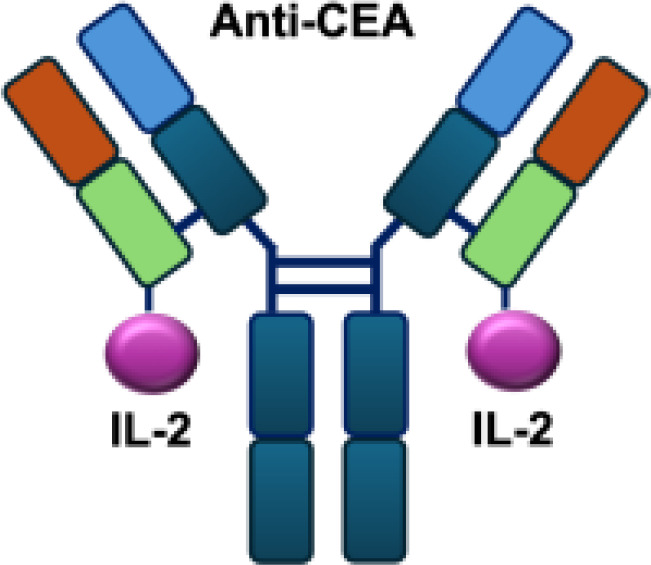	EGFR	B16F10 s.c.; MC38-EGFR5 s.c.; B16-EGFR5 s.c.
KY1043	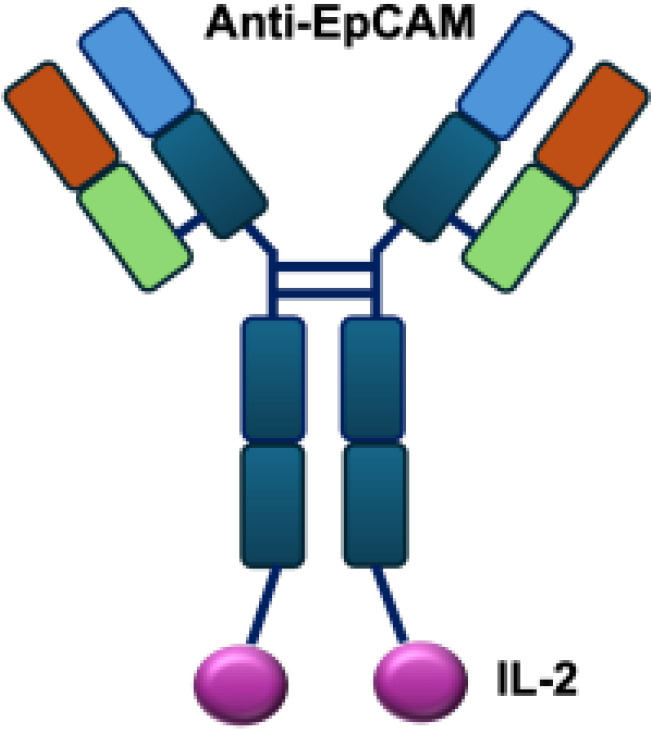	PD-L1	MC38 s.c.; CT26 s.c.; EMT6 s.c.
CEA/FAP/PD1-IL2v	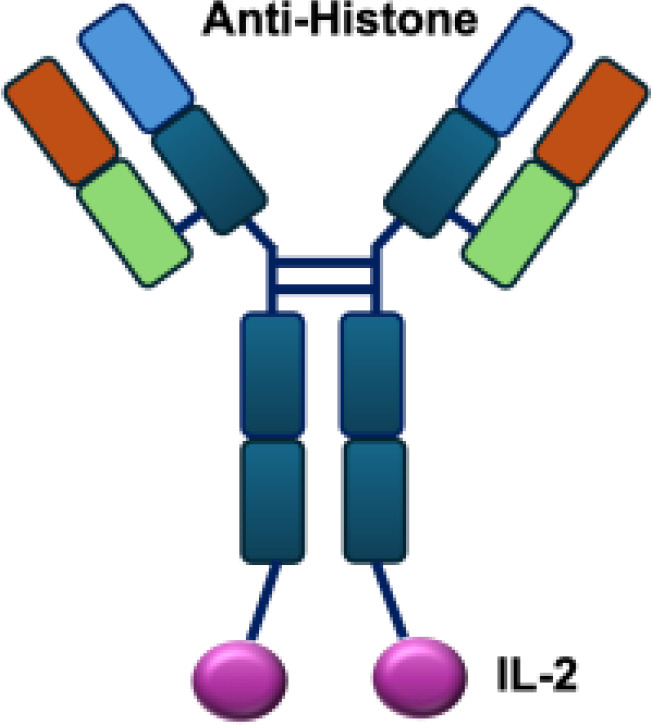	PD-1	E0771/CEA s.c.; MC38/CEA s.c.; B16F10 s.c.; CT26 s.c.; MC38 s.c.
IBI363	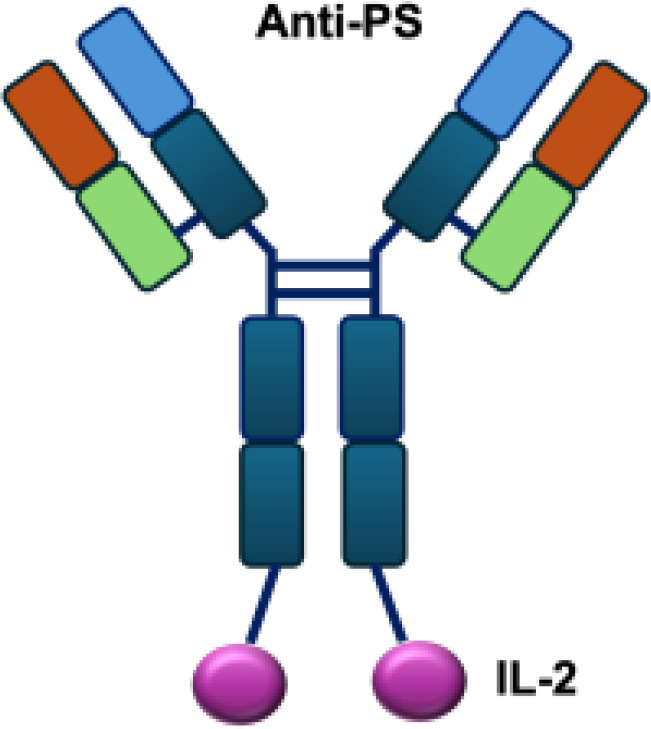	PD-L1	MC38 s.c.; CT26 s.c.
F16-IL2	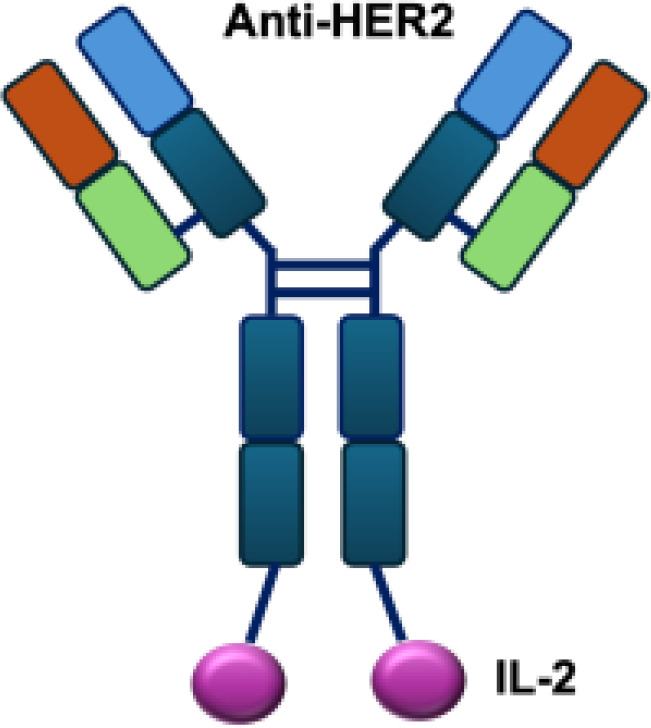	Tnc A1	MDA-MB-231 s.c.; U87MG s.c., i.c.
F8-IL2	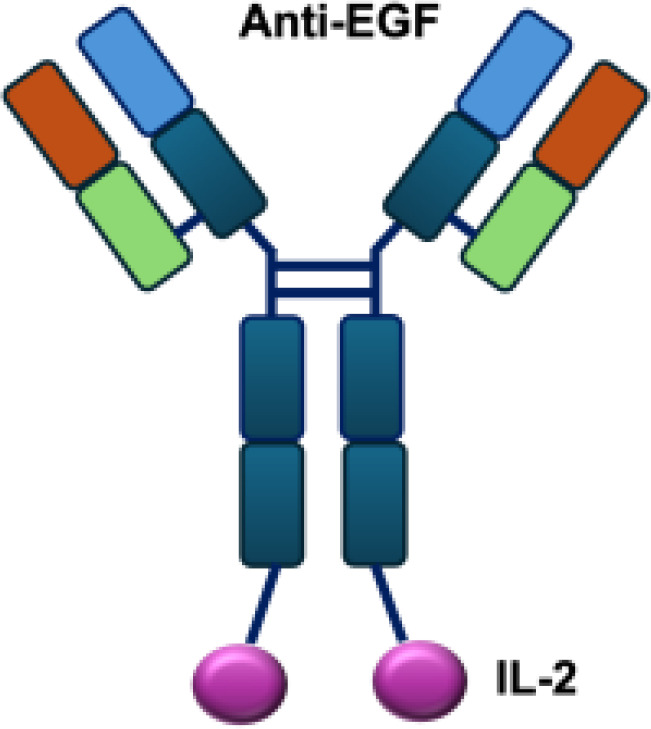	EDA	Caki-1 s.c.; C1498 s.c.; NB4 s.c.; WM1552/5 s.c.; A375M i.v.; K1735M2 s.c.; F9 s.c.; WEHI-163 s.c.
L19-IL2	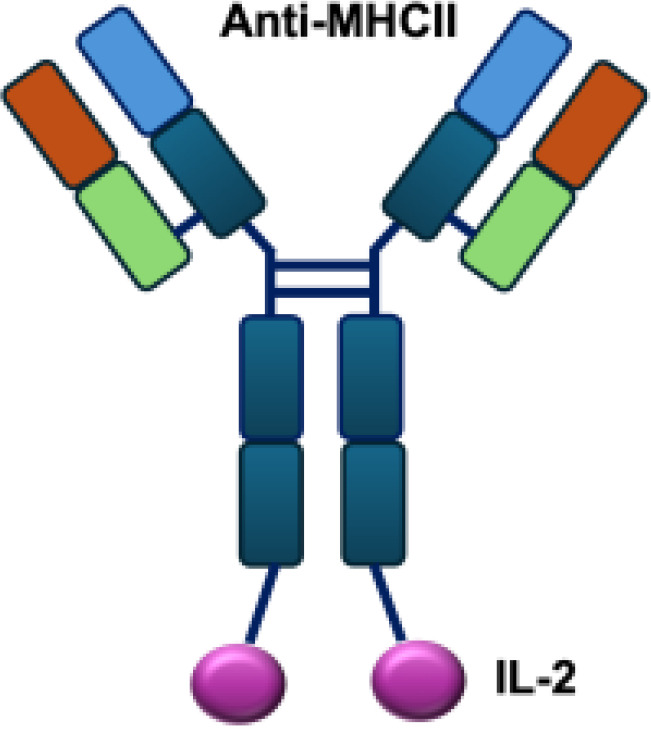	EDB	F9 s.c.; C51 s.c.; N52 s.c.; Ramos s.c., i.v.; DoHH-2 s.c.; CT26 s.c.; K1735M2 s.c.; J558L s.c.; DanG i.p.c.; MiaPaca i.p.c.
Ta99-IL2	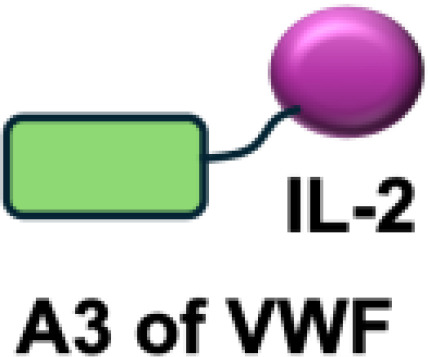	A33	B16F10 s.c.
sm3E-IL2	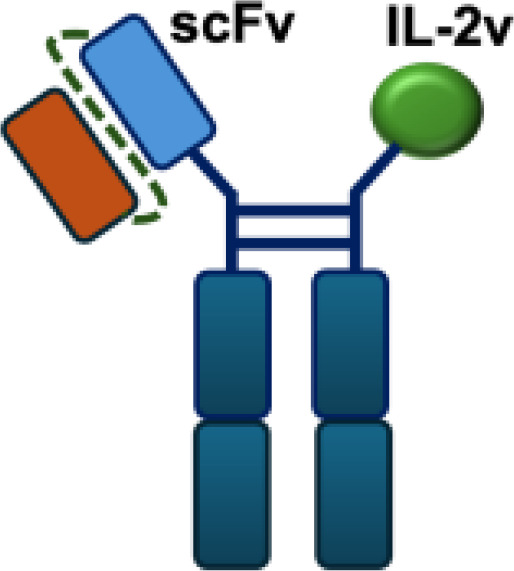	CEA	B16F10 s.c.
KS-IL2	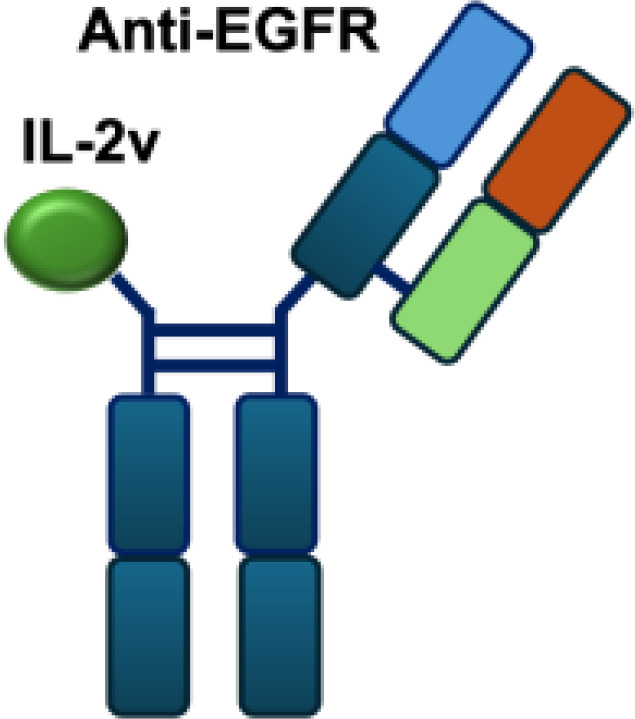	EpCAM	CT26-KSA i.s., i.v., s.c.; PC-3.MM2 i.v.; 4T1-KSA s.c.; LLC-KSA s.c.
NHS-IL2LT	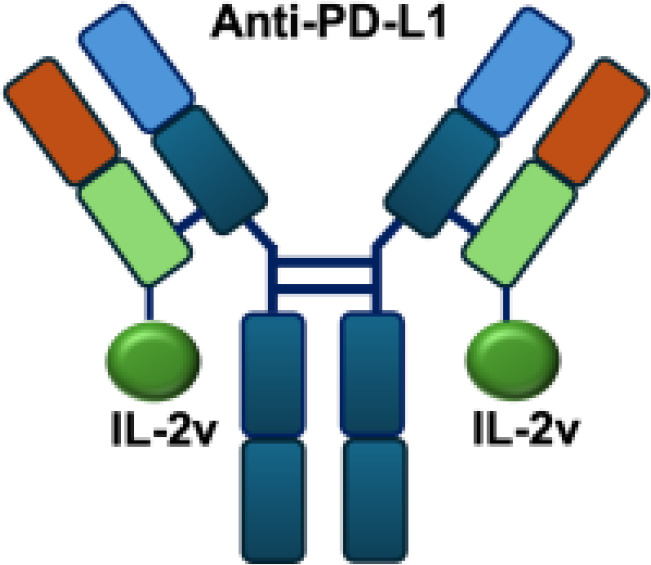	Histone	NX2S i.v.; LCC i.v.
2aG4-IL2	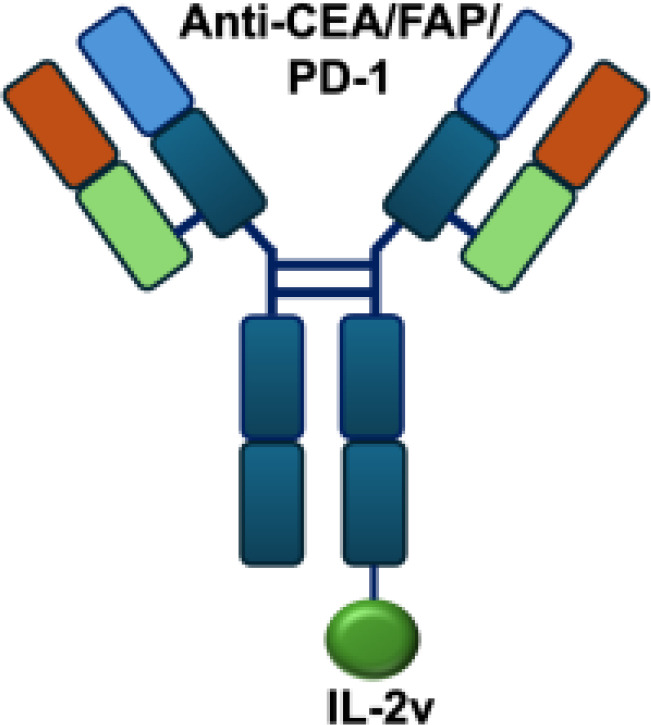	PS	4T1 i.v.
Anti-HER2 IgG3-IL2	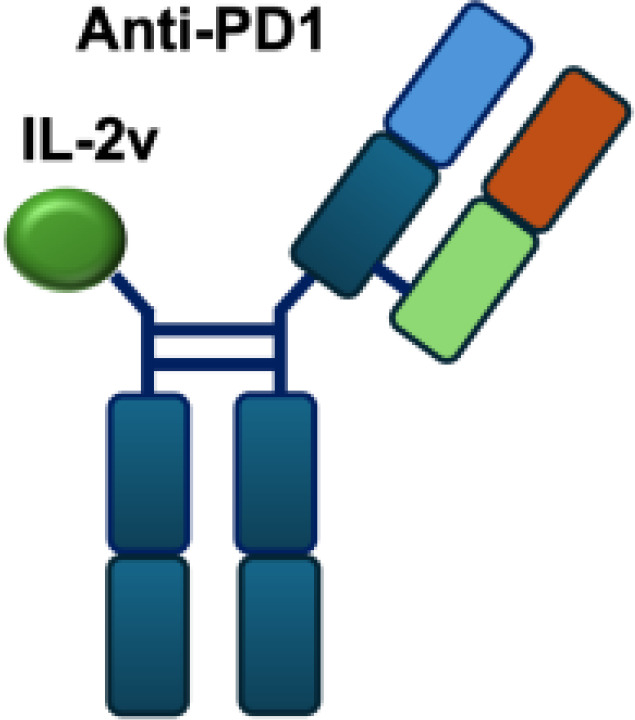	HER2	MC-38 s.c.; MC38-CEA s.c.
ch225-IL2	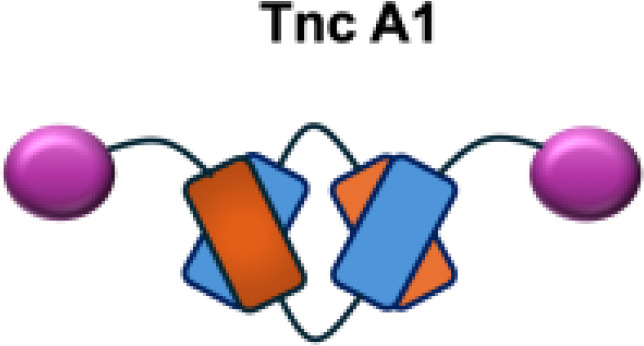	HER2	M24met i.s.
CLL1-IL2	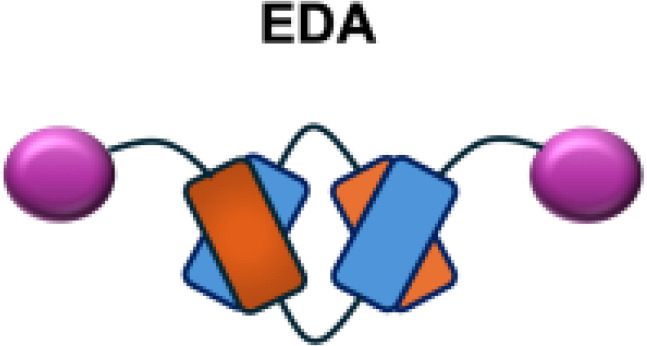	MHCII	ARH-77

s.c., subcutaneous; i.v., intravenous; i.s., intrasplenic; EpCAM, epithelial cell adhesion molecule; HER2, human epidermal growth factor receptor 2; MHCII, major histocompatibility complex class II; PS, phosphatidylserine.

### 3.2 IL-12-based immunocytokines under preclinical research

IL-12 is a heterodimeric cytokine composed of p35 and p40 subunits and produced mainly by antigen-presenting cells such as dendritic cells, macrophages, and B cells. It plays a pivotal role in bridging innate and adaptive immunity by inducing IFN-*γ*secretion, enhancing the cytotoxic activity of NK and CD8^+^ T cells, and promoting the differentiation of naïve CD4^+^ T cells into T helper type 1 (TH1) effectors (45). IL-12 has shown potent anti-tumor and immunostimulatory properties, while its severe systemic toxicity has limited clinical application, even at low doses. Therefore, a variety of IL-12-based targeted delivery approaches have been investigated (35, 46) (**Table 2**).

**Table 2:** Overview of IL−12–based immunocytokines.

Name	Structure	Target	Tumor models
IL12-F8-F8	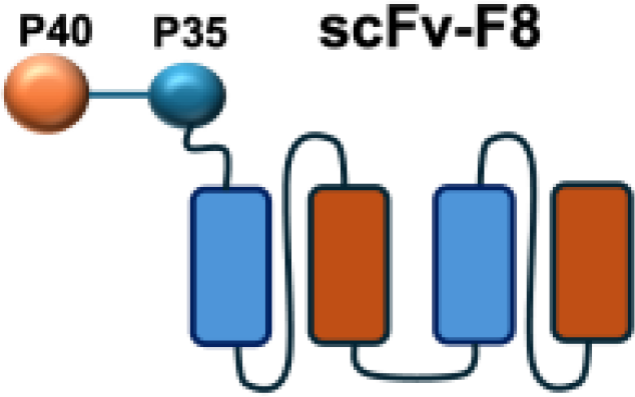	EDA	F9 s.c.; CT26 s.c.; A20 s.c.
L19-IL12	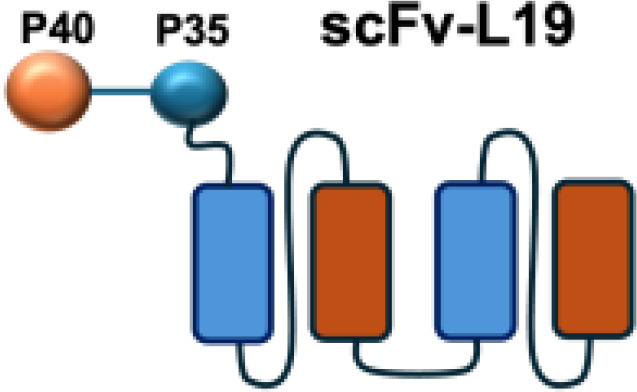	EDA	F9 s.c.
aPD1-mIL12mut2	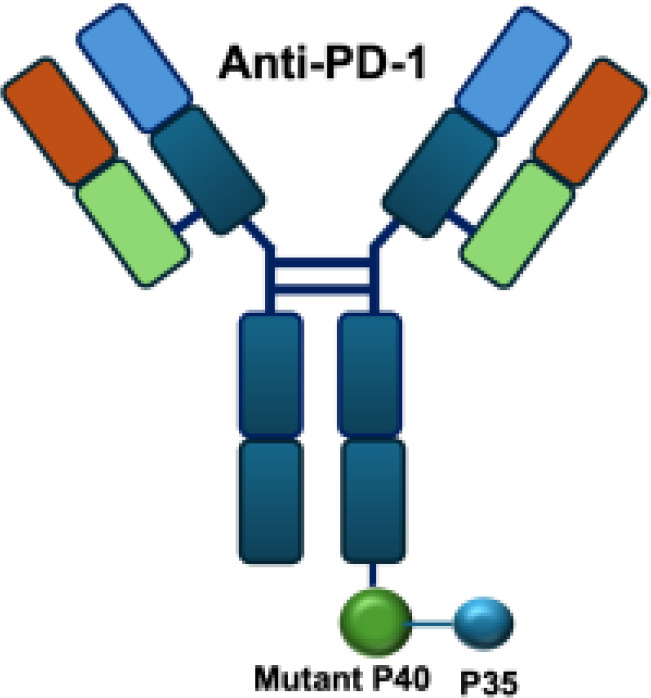	PD−1	MC38 s.c.
NHS−IL12	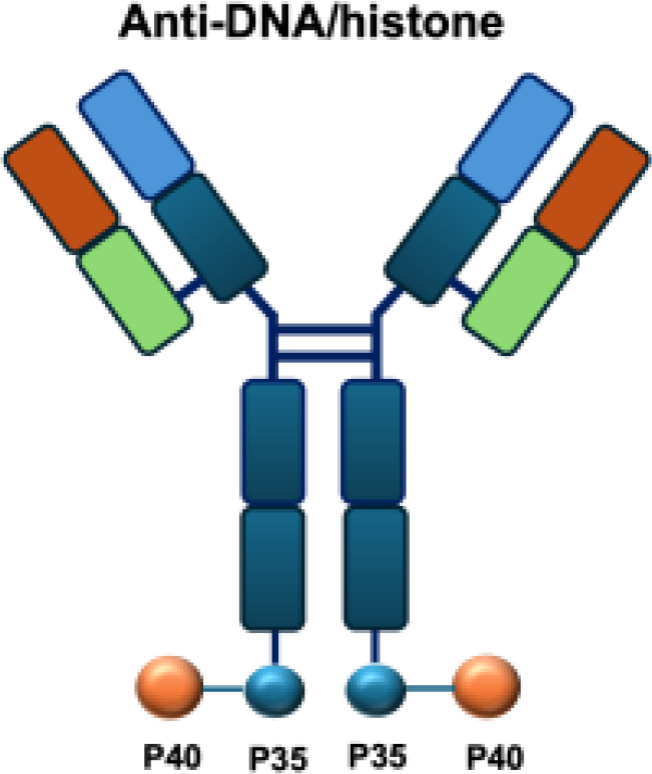	DNA	LS147T s.c.; DU145 s.c.; LLC s.c.; MC38 s.c.; B16 s.c.; MC38/MUC1+ s.c.; PancO2/MUC1+ s.c.; Renca s.c.; PancO2 s.c.; MB49 s.c.
AS1409	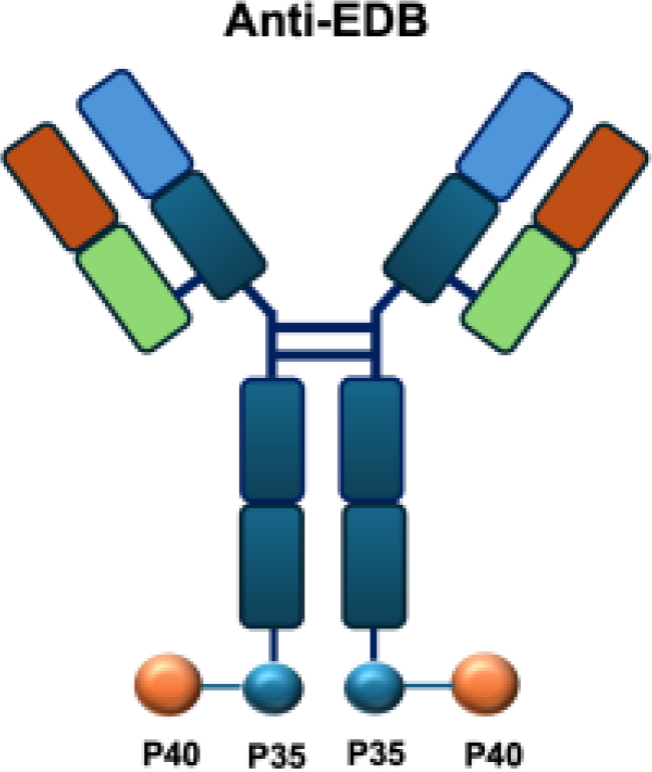	EDB	A375M s.c.

EDA, extra domain A of fibronectin; EDB, extra domain B; PD−1, programmed cell death protein 1; DNA, histone−associated DNA fragments; s.c., subcutaneous.

Among them, IL12-F8-F8 has emerged as a promising candidate (47). This construct is designed as a single polypeptide in which the two IL-12 subunits are linked to the F8 antibody in a single-chain diabody format, an arrangement that facilitates expression compared to more complex architectures. By targeting the alternatively spliced EDA domain of fibronectin, IL12-F8-F8 effectively inhibited tumor growth in models of F9 embryonal carcinoma, CT26 colon carcinoma, and A20 lymphoma. Interestingly, combination with paclitaxel produced synergistic antitumor effects only in the F9 carcinoma model. Mechanistic studies further indicated that NK cells were the principal mediators of the antitumor activity induced by IL12-F8-F8 (47). In addition to IL12-F8-F8, another well-studied IL-12 immunocytokine is L19-IL12, which employs the L19 antibody to target the EDB domain of fibronectin. It was shown to enhance the infiltration and activation of NK and CD8^+^ T cells, and potent tumor growth inhibition, although complete cures are rarely achieved as monotherapy. However, the combination regimens have achieved synergisticeffects and complete tumor regression in immunocompetent mice. Schwager and colleagues showed complete tumor eradications when L19–IL2 was used in combination with CTLA-4 blockade in mouse models with F9 and CT26 tumors.

Interestingly, mice cured from F9 tumors developed new lesions when rechallenged with tumor cells after therapy, whereas mice cured from CT26 tumors were resistant to tumor rechallenge (48). This observation suggests that the antitumor response induced in CT26-bearing mice led to the establishment of immunological memory, consistent with a durable adaptive immune activation. Similar to findings reported for other antibody–cytokine fusion proteins such as L19–IL2 (47), effective treatments were associated with elevated intratumoral levels of pro-inflammatory cytokines (e.g., IL-6, TNF, IL-17) and a marked infiltration of NK cells, macrophages, and CD8^+^ lymphocytes, indicating a coordinated innate and adaptive immune response. In contrast, the lack of protection upon rechallenge in the F9 model may reflect its weaker immunogenicity and limited capacity to induce long-term immune memory (48).

These findings suggest that the therapeutic regimen can elicit durable, tumor-specific immune memory in certain settings, likely mediated by T-cell activation and persistence. Building on this concept, recent studies have shown that tumor-infiltrating PD-11^+^CD8^+^T cells represent an exhausted population characterized by impaired effector function. Therapeutic strategies that target these cells and deliver cytokines may partially reinvigorate their activity and thereby enhance antitumor immunity ()? Tumor-infiltrating PD-1^+^CD8^+^ T cells represent an exhausted T-cell population characterized by impaired effector function. Therapeutic strategies that target these cells and deliver cytokines may partially reinvigorate their activity and thereby enhance antitumor immunity (49). Based on it, The aPD1-mIL12mut2 immunocytokine is engineered by fusing an anti–PD-1 antibody to a low-affinity murine IL-12 mutant. This design enables potent inhibition of tumor growth without systemic toxicity and induces abscopal responses against distant lesions and metastases. Its activity relies on selective engagement of PD-1^+^CD8^+^ T cells in the tumor microenvironment, where cis-binding of the IL-12 mutant via anti–PD-1 enhances bioactivity in an IFN-*γ*–dependent manner (50).

The intratumoral distribution of immunocytokines is often heterogeneous, as antibody moieties primarily target antigens in perivascular regions. This limited penetration can compromise antitumor efficacy by preventing efficient delivery to necrotic tumor areas. To overcome this challenge, the NHS-IL12 immunocytokine was engineered to direct IL-12 to necrotic regions by binding exposed DNA fragments. In preclinical studies, NHS-IL12 showed superior antitumor activity compared with unconjugated IL-12 in models of Lewis lung carcinoma, colon carcinoma, melanoma, and bladder cancer. The therapeutic effect, which prolonged survival, was dependent on NK cells and CD8^+^ T lymphocytes but not on CD4^+^ T cells, and did not confer protection against tumor rechallenge (51, 52). Analysis of the tumor microenvironment revealed a shift toward a pro-inflammatory state, characterized by an increased CD8^+^/CD4^+^ T-cell–to–macrophage/MDSC ratio, reduced intratumoral TGF-*β* levels, and enhanced proliferation and activation of CD4^+^ and CD8^+^ T cells (52). In translational studies, evaluation in canine models of malignant melanoma demonstrated partial responses in 2 of 15 animals, although severe (grade 4–5) toxicities were observed in four cases (53).

In addition, AS1409 is a fusion protein comprising IL-12 linked to the humanized antibody BC1, which targets the EDB of fibronectin in the tumor vasculature. In preclinical studies, AS1409 demonstrated selective tumor localization and potent antitumor activity across several xenograft models. Treatment enhanced local immune activation, including the induction of interferon-*γ* and proliferation of cytotoxic T lymphocytes, while reducing systemic exposure compared with recombinant IL-12. These findings provided the rationale for its subsequent evaluation in early-phase clinical trials (54, 55).

### 3.3 TNF-based immunocytokines under preclinical research

L19–TNF*α* is an immunocytokine in which a scFv specific for the EDB domain of fibronectin is genetically fused to TNF (**Table 3**). Owing to the natural trimerization of TNF, the fusion protein assembles into a homotrimeric structure, presenting three L19 scFvunits together with one bioactive TNF trimer. This multivalent configuration enhances binding avidity and supports selective accumulation at the tumor site, thereby limiting off-target toxicity (56). In the preclinical studies, the combination of L19-TNF and L19–IL2 indicated complete remissions when administered in a single intratumoral injection, whereas the monotherapy did not lead to cures (48). Mechanistically, the combination of L19-TNF*α* and L19-IL2 eradicated tumors in J558L myeloma BALB/c mice likely via TNF*α*-induced tumor necrosis and L19-TNF*α*/L19-IL2-mediated local cellular immune reactions (57).

**Table 3:** Overview of TNF−based immunocytokines.

Name	Structure	Target	Tumor models
F8−TNF	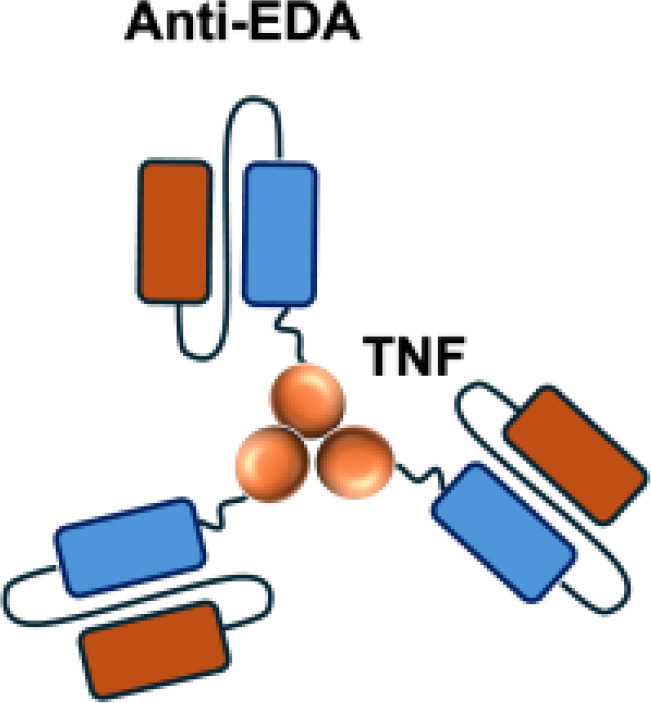	EDA	WEHI−164 s.c.; Sarcoma 180 s.c.
L19−TNF	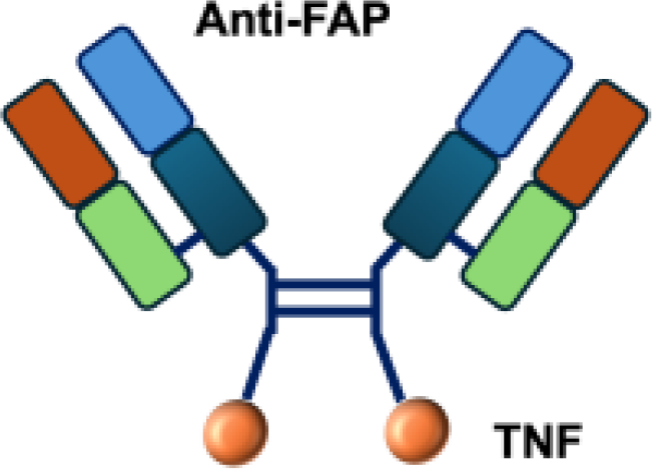	EDB	F9 s.c.; WEHI−164 s.c.; C51 s.c.; N2A s.c.; NIE−115 s.c.; K1735M2 s.c.; J558L s.c.
scFvMEL−TNF	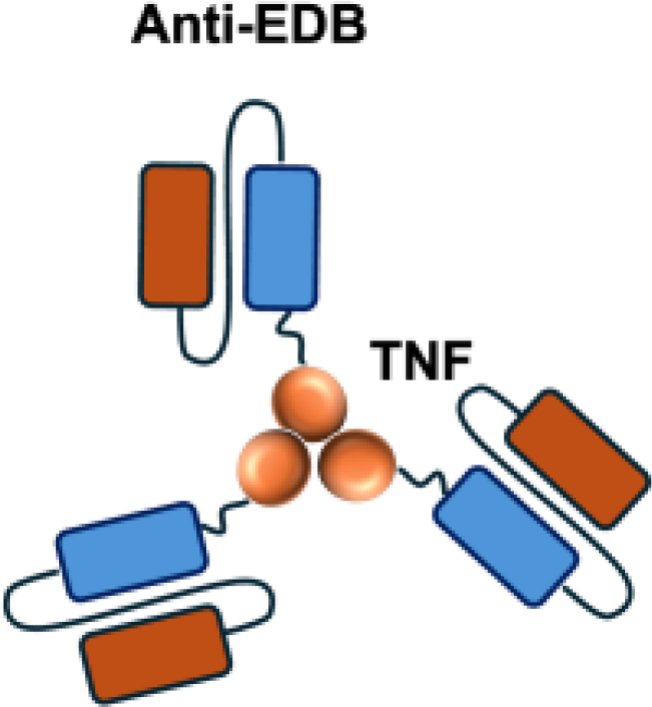	EDB	A375 s.c.
MFE23−TNF	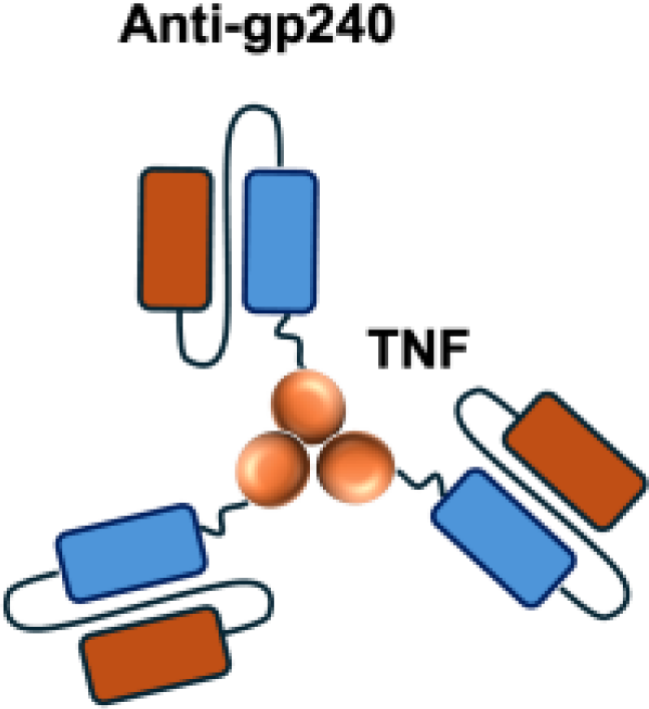	CEA	LS174T s.c.
G250−TNF	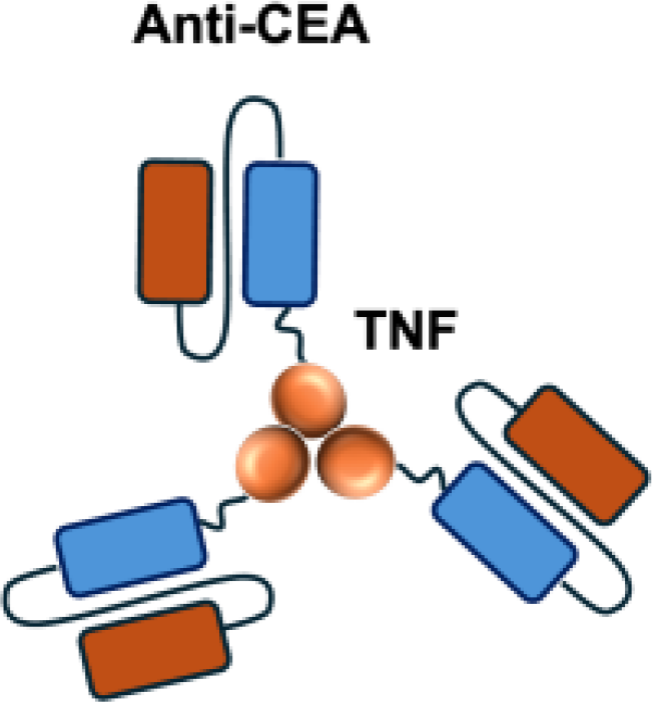	CAIX	NU−12 s.c.; SK−RC17/52 s.c.
TNF−TNT3	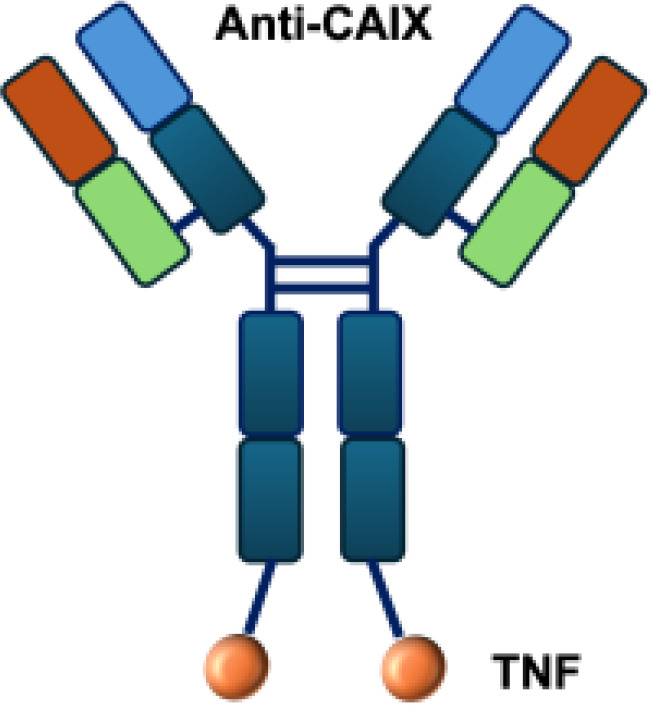	DNA	LS174T s.c.
TNF−FuP	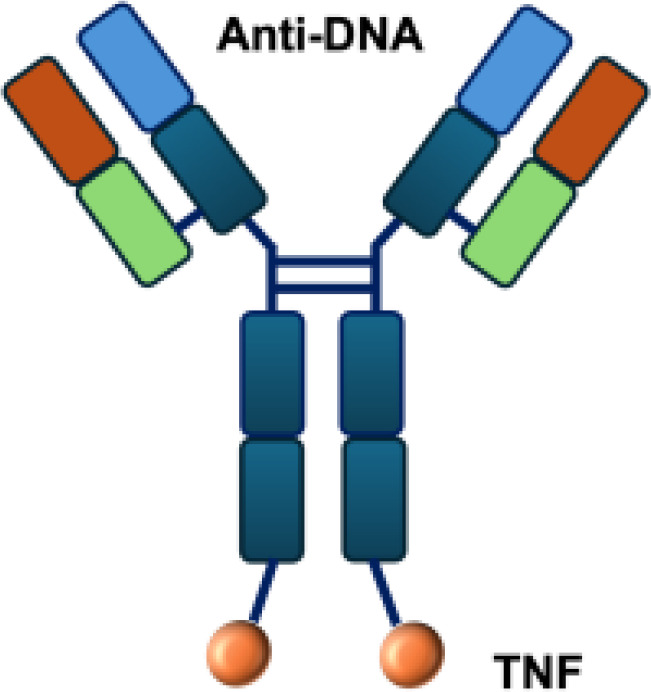	EGFR	BLM s.c.
TNF−B1	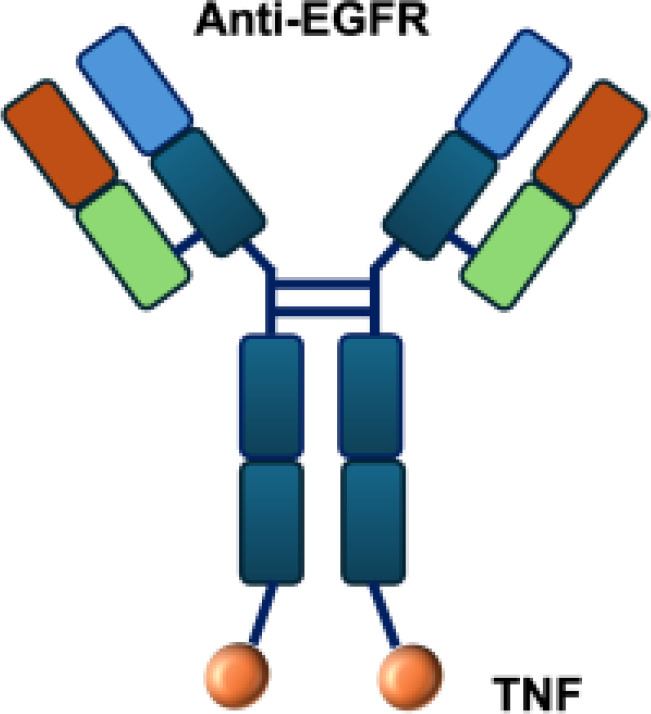	LeY	MCF−7 s.c.
ZME/TNF	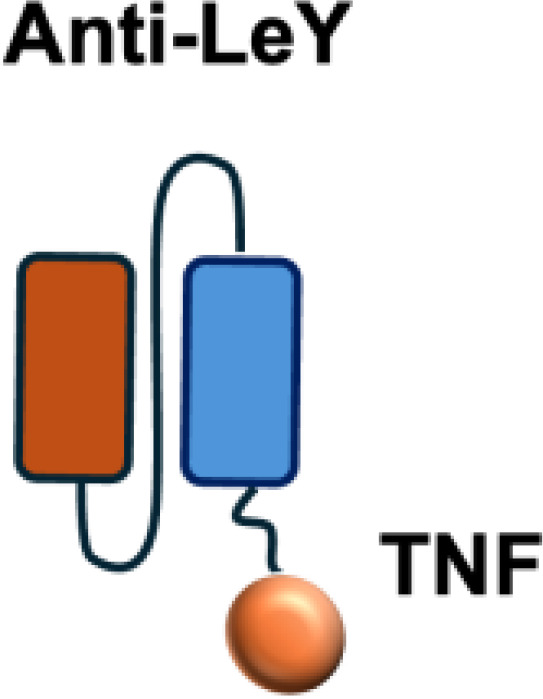	gp240	A375 s.c.
FAP−TNF	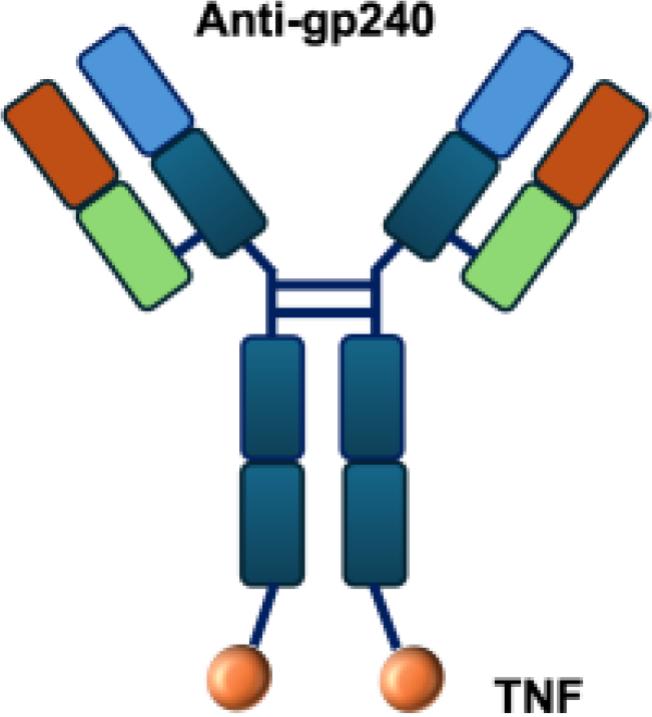	FAP	HT1080−FAP+ s.c.

EDA, extra domain A; EDB, extra domain B; CEA, carcinoembryonic antigen; CAIX, carbonic anhydrase IX; DNA, histone−associated DNA fragments; EGFR, epidermal growth factor receptor; LeY, Lewis Y antigen; gp240, melanoma−associated chondroitin sulfate proteoglycan; FAP, fibroblast activation protein; s.c., subcutaneous.

Extensive investigations have also been carried out with diabody formats fused to TNF*α*. One such construct, scFv23-TNF, was engineered to target the human epidermal growth factor receptor 2 (HER-2), which is overexpressed in approximately 30% of breast cancers. scFv23-TNF*α* demonstrated potent cytotoxicity against HER-2 overexpressing tumor cells. In these studies, treatment of HER2/neu–overexpressing SKBR-3-LP breast cancer cells with scFv23–TNFα led to a 5- to 7-fold increase in TNFαreceptor-1 (TNFR1) expression, downregulation of Akt phosphorylation, and subsequent activation of the apoptotic cascade through caspase-8 and caspase-3 cleavage. The reduction in Akt phosphorylation reflects inhibition of the PI3K/Akt survival pathway, thereby sensitizing TNF-resistant HER2-positive tumor cells to apoptosis (58).

## 4 Clinical development of immunocytokines

Mutant IL-2–based immunocytokines have advanced into clinical trials. Because CD25 is highly expressed on Tregs, low doses of WT IL-2 preferentially expand this immunosuppressive population. To overcome this limitation, engineered IL-2 variants were developed that selectively bind the intermediate-affinity CD122/CD132 receptor complex, thereby avoiding Tregs activation. Roche generated CEA-IL2v and FAP-IL2v by fusing IL-2 variants to antibodies targeting carcinoembryonic antigen (CEA) and fibroblast activation protein (FAP), respectively (59).

To ensure site-specific cytokine attachment, Roche employed knob-into-hole technology, an antibody-engineering strategy in which complementary mutations (‘knob’ and ‘hole’) are introduced into the CH_3_ domains of two heavy chains to favor heterodimer formation between them, ensuring that one heavy chain carries the IL-2 fusion while the other does not (60). Compared with their wild-type counterparts, both CEA-IL2v and FAP-IL2v demonstrated improved tumor-to-organ ratios and enhanced pharmacokinetic profiles in preclinical studies. Multiple clinical trials have been initiated to assess CEA-IL2v and FAP-IL2v, both as monotherapies and in combination with immune checkpoint inhibitors (atezolizumab, NCT02350673, NCT03063762, NCT03386721), trastuzumab (anti-HER2; NCT02627274), or cetuximab (anti-EGFR; NCT02627274).

IL-2R*α* biased agonists have been shown to significantly expand tumor-specific CD8^+^ T cells (TSTs) and PD-1^+^CD25^+^CD8^+^ tumor-infiltrating lymphocytes (TILs) (61), although they may also promote Tregs proliferation. A PD-1 antibody fused to an engineered IL-2, termed IBI363, was designed to simultaneously block the PD-1 checkpoint and revitalize exhausted TSTs via its IL-2 mutant domain. In phase I clinical studies, IBI363 demonstrated promising antitumor activity in patients with advanced non–small cell lung cancer (NSCLC) and melanoma (NCT05290597; NCT05460767), with a favorable tolerability profile (62). In addition, themaximum tolerated dose (MTD) and pharmacokinetics of NHS-IL12 were evaluated in patients with advanced epithelial or mesenchymal tumors in a phase I trial (63). Although no objective tumor responses were observed, 5 of 59 patients achieved durable stable disease lasting 6 to 30 months. NHS-IL12 was well tolerated up to 16.8 μg/kg, which was established as the recommended phase II dose for subsequent combination studies. In a phase I/II clinical trial (NCT04633252), NHS-IL12 combined with docetaxel, a tumor necrosis–inducing standard-of-care agent, was investigated in metastatic prostate cancer. In a separate phase Ib study (NCT02994953), NHS-IL12 in combination with avelumab (anti–PD-L1) demonstrated clinical activity. During dose escalation, the regimen was well tolerated and the MTD was not reached. Notably, two patients with advanced bladder cancer achieved durable complete responses; however, in the subsequent expansion cohort, no objective responses were observed among 16 patients with advanced urothelial carcinoma (UC) (63).

In murine models of GL-261 glioblastoma and SMA-560 astrocytoma, L19-IL12 efficiently localized to brain tumors and achieved cure rates of 40% and 29%, respectively (64). On the basis of these findings, a phase I clinical trial is currently evaluating L19-IL12 in patients with advanced or metastatic solid tumors who have previously received immune checkpoint inhibitors (NCT04471987). The study is designed to assess safety, pharmacokinetics, and to determine the MTD in order to establish a recommended dose for future studies.

TNF exerts dual functions in the immune system, displaying both pro-inflammatory and immunosuppressive activities. TNF was originally identified for its potent cytotoxic and immune-stimulatory effects against tumors. However, more recent studies have uncovered a paradoxical role in the tumor microenvironment, where TNF can also act as an immunosuppressive cytokine. In melanoma, for instance, TNFR1-dependent TNF signaling was shown to limit CD8^+^ TIL accumulation and to promote PD-L1 andTIM-3 expression, thereby reducing the efficacy of anti–PD-1 therapy. Notably, TNF blockade synergized with PD-1 inhibition, providing a rationale for combined anti-TNF and immune checkpoint blockade strategies (65).

Based on its favorable antitumor activity and pharmacokinetic profile in preclinical models, L19-TNF (Fibromun) the only TNF-based immunocytokine entered into clinical trials. In a phase I/II study, L19-TNF monotherapy was well tolerated at doses up to 13μg/kg, with only mild chills, nausea, and vomiting reported, and the MTD was not reached. Although no objective tumor responses were observed, disease stabilization without progression was achieved, suggesting the potential for further dose escalation in combination regimens (66). Notably, in patients undergoing isolated limb perfusion, the combination of L19-TNF with melphalan induced objective responses in 89% of cases, including complete responses in 5 of 10 patients, four of which were maintained for at least 12 months (67).

Fibromun has now progressed to phase III clinical trials, including a study in combination with doxorubicin for patients with metastatic soft-tissue sarcoma (EudraCT number 2016–003239-38) (68), and another in combination with L19-IL2 (Darleukin) for patients with fully resectable stage IIIB/C melanoma (ClinicalTrials.gov identifier NCT02938299; EudraCT number 2015–002549-72) (69). Furthermore, intratumoral administration of L19-TNF together with L19-IL2 in inoperable stage IIIC and IVM1a metastatic melanoma induced systemic antitumor immunity, resulting in complete responses in 53.8% of 13 non-injected lesions (70).

## 5 Approaches to reduce systemic cytotoxicity

Despite the promising antitumor activity of immunocytokines, their clinical translation has been hampered by dose-limiting systemic toxicities resulting from cytokine activity in healthy tissues. For example, clinical trials with IL2 and TNF based immunocytokines (such as L19–IL2, L19–TNF, and NHS–IL12) have reported flu-like symptoms, hypotension, and liver enzyme elevations at therapeutic doses, limiting therapeutic alternations (48). To overcome this challenge, several engineering strategies have been developed to enhance tumor selectivity while reducing off-target toxicity. These approaches primarily include the use of cytokine mutants, Fc point mutations, and Conditionally-activated immunocytokine prodrugs.

### 5.1 Reduction of payload‐mediated toxicity

The cytokine payloads of immunocytokines contribute to dose-limiting toxicities. Several strategies have been developed to overcome these limitations. Lowering the affinity of cytokines for their cognate receptors can attenuate systemic activation, prolong circulation time, and enhance antibody-mediated accumulation at the tumor site (71). For example, several IL-2 variant–based immunocytokines have been engineered with reduced affinity for the *βγ* receptor complex. These variants exhibit minimal systemic activity (72), which allows administration at higher doses. In addition, fusion proteins combining anti–PD-1 antibodies with mutant cytokines, such as IL-15 and IFN-*α* with attenuated receptor binding, have demonstrated enhanced antitumor efficacy in murine models (73).

Furthermore, certain cytokines achieve biological activity only in their multimeric form rather than as monomers. Exploiting this property, the L19–TNF-*α* immunocytokine was designed to mitigate systemic toxicity when administered intravenously (48). While TNF-*α* is active as a homotrimer, IL-12 functions as a heterodimer composed of the p35 and p40 subunits. By fusing each subunit to antibodies for targeted delivery into the tumor microenvironment, functional IL-12 activity can be restored upon dimer assembly (74.

### 5.2 Conditionally-activated immunocytokine prodrugs

**Figure 3:**
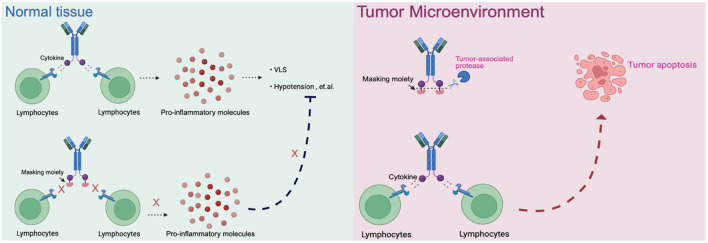
The schematic of prodrugs in normal tissue and tumor microenvironment. Normal tissue: Cytokines or immunocytokines without a masking moiety can active lymphocytes systemically, leading to widespread release of pro inflammatory mediators and dose-limiting toxicities. When masked, cytokine activity is blocked, preventing unwanted immune activation in healthy tissues. Tumor microenvironment: Protease-rich conditions allow cleavage of the masking moiety, restoring cytokine activity. The locally released cytokine activates tumor-infiltrating lymphocytes, driving antitumor immune respoinses and promoting tumor apoptosis.

Prodrug-based strategies represent a promising approach to improve the safety profile of immunocytokines while preserving antitumor activity. The core principle of this design is to attach an epitope-masking moiety to the antibody through a protease-cleavable linker, enabling selective activation in the tumor microenvironment where proteases are abundantly expressed (**Figure 3**). WTX-124, an IL-2 prodrug consisting of native human IL-2 linked to a Fab antibody fragment (inactivation domain) and a single-domain antibody targeting human albumin (half-life extension domain), is currently under evaluation in a phase I clinical trial (NCT05479812) by Werewolf Therapeutics (75). Similarly, IL-15–based immunocytokines such as PEG-masked IL-15, ASKG915, and LH05 have demonstrated the potential for tumor-selective activation and reduced systemic toxicity in preclinical studies (76; 77).

### 5.3 Silencing Fc functions of antibody by point mutations

The toxicity of immunocytokines is mainly attributed to their cytokine portion, while their antibody component also has potential implications for safety. In their antibody parts, the antigen binding regions of immunocytokines can cause the normal tissuedamage due to On-target Off-tumor binding, although the low expression of antigens in normal cells. In addition, Fc region has a significant implication for the cytotoxicity in normal tissues (22). Fc receptors such as Fc*γ*Rs, FcRn and C-type lectin receptors (CLRs), mainly expressed on the innate and adaptive immune cells, can contribute to target-independent trafficking of immunocytokines in normal tissues (78). A potential solution is to use an antibody fragment that lacks the Fc region, such as scFv and F(ab)_2 fragments, but with the compensation of the reduced half-life and retention at the tumor site. In order to overcome this limitation, introduction of mutations in Fc regions provides an alternative approach. For instance, the engineering Fc including KD033 (L234A/L235A mutation in Fc region), as well as LH01(a N297A mutation), both of them have been shown to enhance efficacy by minimizing ADCC to enhance the safety of immunocytokines (79).

## 6 Summary and future directions of immunocytokines

### 6.1 Opportunities for combination therapy

The therapeutic potential of immunocytokines can be greatly enhanced through rational combination strategies that exploit their unique ability to remodel the TME. Besides stimulating antitumor immunity, proinflammatory cytokines such as TNF*α* transiently enhance vascular permeability and endothelial activation, thereby facilitating the intratumoral accumulation of complementary agents (35; )?. Based on this property, immunocytokines is able to be used in combination with other therapeutic methods, including chemotherapy, immune checkpoint blockade, adoptive cell transfer (ACT) (??^9^, ^55^).

Future research may focus on mechanistically synergistic combinations. For instance, immunocytokines that selectively activate effector T or NK cells within the TME could be combined with agents that promote immune response, such as checkpoint inhibitors or macrophage reprogramming drugs. Recent preclinical studies were consistent with this principle, such as PD-1/IL-2v fusions combined with anti-PD-L1 antibodies which enhanced the expansion of stem-like CD8^+^ T cells and reprogrammed macrophages toward a proinflammatory phenotype ()?, while anti-PD-L1-IL15 immunocytokine prodrugs (e.g., LH05) synergized with oncolytic viruses to strengthen CD8^+^ T cell effector functions ()?.

Clinically, the encouraging efficacy of N-803 plus BCG in non-muscle-invasive bladder cancer (NMIBC) supports the translational feasibility of such combinations ()?. In addition, due to the pivotal role of IL-15 in NK cells proliferation and memory formation, IL-15 based immunocytokines are particularly well suitable for combination with NK cell therapies to achieve durable antitumor immunity ()?,?. Therefore, the next generation of immunocytokine therapy may lie in systematically designing combination regimens based on quantitative modeling of cytokine dynamics, immune cell kinetics, and TME remodeling. Such integrative approaches could enable personalized selection of immunocytokine partners and dosing regimens, maximizing efficacy while minimizing toxicity.

### 6.2 Improving dose-limiting side effects

Systemic toxicity remains the main barrier for the widespread clinical application of immunocytokines. Future research could focus on precisely controlling cytokine activity in space and time. Prodrug or “masked” cytokine formats already allow conditional activation within TME by exploiting features such as protease abundance, acidic pH, or altered redox potential (?^76^; )?. However, tumoral heterogeneity in these features can lead to variable activation, underscoring the need for next-generation designs with tunable activation thresholds that respond dynamically to the TME. Multilayered control systems integrating both affinity modulation and conditional activation may offer superior safety and predictability. Engineering cytokines with selectively weakened receptor binding could minimize peripheral signaling, while proteaseor pH dependent unmasking ensures potent activity within tumors ()??. Incorporating computational modeling and artificial intelligence guided protein design could further enable the rational optimization of such constructs, balancing pharmacodynamic potency and pharmacokinetic behavior ()??.

Another promising direction is the development of adaptive or switchable cytokines, capable of reversible activation via external cues such as small molecules, light, or ultrasound (??^76^; )?. These platforms could allow clinicians to fine tune cytokine exposure in real time, thereby mitigating dose limiting adverse effects. Ultimately, future immunocytokines will likely combine precision targeting, conditional activation, and controllable signaling intensity, transforming cytokine therapy from a blunt systemic tool into a finely adjustable immunomodulatory instrument.

### 6.3 Selection of different payloads for immunocytokines

Harnessing multiple cytokine pathways within a single immunocytokine offers a promising route to achieve broader and more durable antitumor responses. Preclinical and clinical evidence indicates that combining cytokines with complementary mechanisms such as IL-2 with TNF*α*, IL-12, can produce synergistic immune activation and improved tumor control (48, 80; )?. However, conventional combinational administration of distinct cytokine productspresent dose optimization and pharmacokinetic challenges, as each cytokine exhibits unique potency, half-life, and tissue distribution (56).

To overcome these limitations, next generation dual and multiple cytokine immunocytokines are being engineered to deliver two payloads in a single, coordinated molecule, thereby simplifying clinical development and ensuring synchronized pharmacodynamics (?^81^). The relative activity of each cytokine must be carefully balanced to avoid antagonistic signaling or systemic toxicity. For example, IL-2 fusions often require doses over tenfold higher than TNF fusions to achieve comparable potency (70). To address this, the Neri team designed a potency matched IL-2–F8–TNF mutant construct incorporating a TNF mutein with reduced activity, which produced superior antitumor efficacy and improved tolerability in murine models ()??.

The field is moving toward modular immunocytokines capable of delivering multiple cytokines with programmable ratios and controlled release. Advances in computational protein design and synthetic linker engineering may enable precise tuning of payload balance and spatial arrangement, allowing coordinated activation of immune pathways while preserving the specificity and safety of a single targeted therapeutic.

## 7 Conclusions

Immunocytokines combine the targeting ability of antibodies with the pro-inflammatory power of cytokines. This approach can focus treatment on tumors while reducing dose limited side effects. Recent progress in protein engineering has led to several promising candidates now being tested in clinical trials. However, there are challenges, such as the effectiveness of tumor eradication, attenuating the side effects, improving the pharmacokinetics, and optimizing homogeneity of the drug distribution in tumors. The smarter cytokine designs, the combination therapies, and the innovation are likely to define the next generation of immunocytokines for cancer immunotherapy.
