# Supplementary material for: Immunocytokines in cancer immunotherapy: opportunities and challenges
Source: Front Oncol. 2026 Jan 6;15:1716612. doi: 10.3389/fonc.2025.1716612 (PMC12817307; doi:10.3389/fonc.2025.1716612)
Supplement: Supplementary file 1 [file DataSheet1.pdf]

Supplementary Material

1 SUPPLEMENTARY DATA

Supplementary Material should be uploaded separately on submission. Please include any supplementary data, figures and/or tables. All supplementary files are deposited to FigShare for permanent storage and receive a DOI.

Supplementary material is not typeset so please ensure that all information is clearly presented, the appropriate caption is included in the file and not in the manuscript, and that the style conforms to the rest of the article. To avoid discrepancies between the published article and the supplementary material, please do not add the title, author list, affiliations or correspondence in the supplementary files.

2 SUPPLEMENTARY TABLES AND FIGURES

2.1 Tables

Table S1. Overview of IL-2–based immunocytokines (part I).

Name	Structure	Target	Tumor models
Anti-CEA-IL2	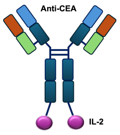	CEA	E0771/CEA<br>MC38/CEA
CBD-IL2	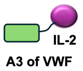	Collagen	B16F10 melanoma<br>CT26 colon carcinoma<br>MMTV-PyMT breast cancer
PD-1-laIL2	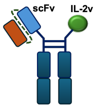	PD-1	MC38, B16F10
Erb-sumIL2	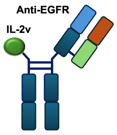	EGFR	B16F10 s.c.<br>MC38-EGFR5 s.c.<br>B16-EGFR5 s.c.
KY1043	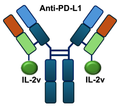	PD-L1	MC38 s.c.<br>CT26 s.c.<br>EMT6 s.c.
CEA/FAP/PD1-IL2v	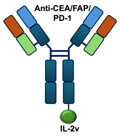	PD-1	E0771/CEA s.c.<br>MC38/CEA s.c, B16F10 s.c.<br>CT26 s.c., MC38 s.c.<br>B16F10 s.c.
IBI363	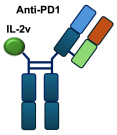	PD-L1	MC38 s.c., CT26 s.c.
F16-IL2	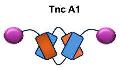	Tnc A1	MDA-MB-231 s.c., U87MG s.c., i.c.
F8-IL2	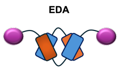	EDA	Caki-1 s.c., C1498 s.c., NB4 s.c.<br>WM1552/5 s.c., A375M i.v.<br>K1735M2 s.c., F9 s.c.<br>WEHI-163 s.c.

Table S2. Overview of IL-2–based immunocytokines (part II)

Name	Structure	Target	Tumor models
L19-IL2	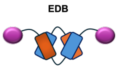	EDB	F9 s.c., C51 s.c., N52 s.c.<br>Ramos s.c., i.v.<br>DoHH-2 s.c.<br>CT26 s.c.<br>K1735M2 s.c., J558L s.c.<br>DanG i.p.c., MiaPaca i.p.c.
Ta99-IL2	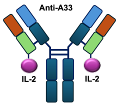	A33	B16F10 s.c.
sm3E-IL2	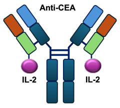	CEA	B16F10 s.c.
KS-IL2	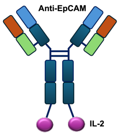	EpCAM	CT26-KSA i.s., i.v., s.c., PC-3.MM2 i.v., 4T1-KSA s.c., LLC-KSA s.c.
NHS-IL2LT	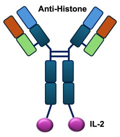	Histone	NX2S i.v., LCC i.v.
2aG4-IL2	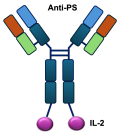	PS	4T1 i.v.
Anti-HER2 IgG3-IL2	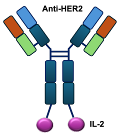	HER2	MC-38 s.c.<br>MC38-CEA s.c.
ch225-IL2	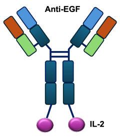	HER2	M24met i.s.
CLL1-IL2	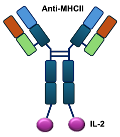	MHCII	ARH-77

Abbreviations: s.c., subcutaneous; i.v., intravenous; i.s., intrasplenic; EpCAM, epithelial cell adhesion molecule; HER2, human epidermal growth factor receptor 2; MHCII, major histocompatibility complex class II; PS, phosphatidylserine.

Table S3. Overview of IL-12-based immunocytokines

Name	Structure	Target	Tumor models
IL12-F8-F8	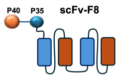	EDA	F9 s.c., CT26 s.c., A20 s.c.
L19-IL12	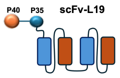	EDA	F9 s.c.
aPD1-mIL12mut2	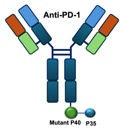	PD-1	MC38 s.c.
NHS-IL12	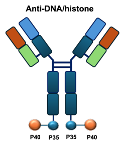	DNA	LS147T s.c., DU145 s.c.<br>LLC s.c., MC38 s.c.<br>B16 s.c., MC38/MUC1+ s.c.<br>PancO2/MUC1+ s.c., Renca s.c.<br>PancO2 s.c., MB49 s.c.
AS1409	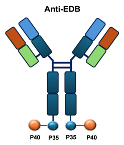	EDB	A375M s.c.

Abbreviations: EDA, extra domain A of fibronectin; EDB, extra domain B of fibronectin; PD-1, programmed cell death protein 1; DNA, histone-associated DNA fragments in necrotic tissue; s.c., subcutaneous.

Table S4. Overview of TNF-based immunocytokines

Name	Structure	Target	Tumor models
F8-TNF	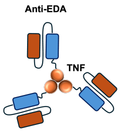	EDA	WEHI-164 s.c., Sarcoma 180 s.c.
L19-TNF	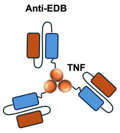	EDB	F9 s.c., WEHI-164 s.c.<br>C51 s.c., N2A s.c.<br>NIE-115 s.c., K1735M2 s.c.<br>J558L s.c.
scFvMEL-TNF	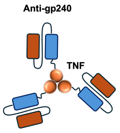	EDB	A375 s.c.
MFE23-TNF	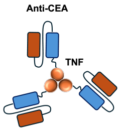	CEA	LS174T s.c.
G250-TNF	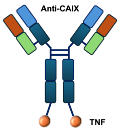	CAIX	NU-12 s.c., SK-RC17/52 s.c.
TNF-TNT3	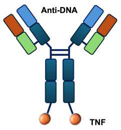	DNA	LS174T s.c.
TNF-FuP	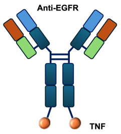	EGFR	BLM s.c.
Christ et al. (2001)			
TNF-B1	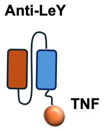	LeY	MCF-7 s.c.
ZME/TNF	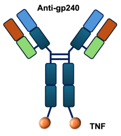	gp240	A375 s.c.
FAP-TNF	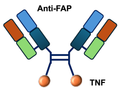	FAP	HT1080-FAP+ s.c..

Abbreviations: EDA, extra domain A of fibronectin; EDB, extra domain B of fibronectin; CEA, carcinoembryonic antigen; CAIX, carbonic anhydrase IX; DNA, histone-associated DNA fragments in necrotic tissue; EGFR, epidermal growth factor receptor; LeY, Lewis Y antigen; gp240, melanoma-associated chondroitin sulfate proteoglycan; FAP, fibroblast activation protein; s.c., subcutaneous.

2.2 Figures

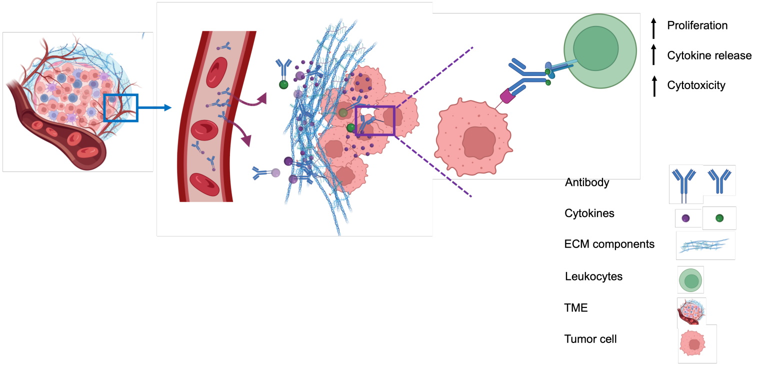

Figure S1. Antibody-mediated delivery of immunocytokines to the tumor microenvironment. Antibodies direct cytokines to tumor-associated antigens, promoting immune cell proliferation, cytokine release, and cytotoxicity, while reducing systemic toxicity.

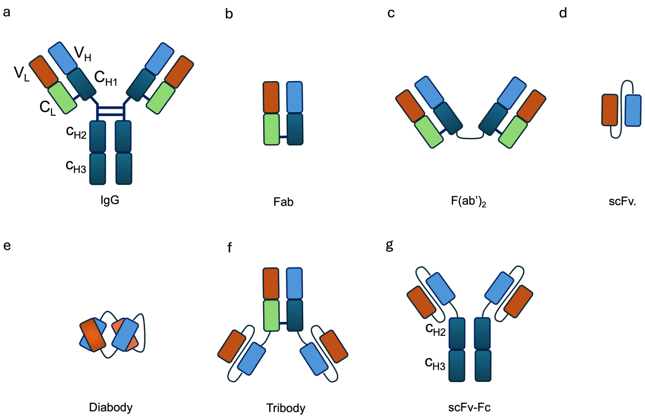

Figure S2. Schematic representation of different antibody formats used in immunocytokine design. (a) IgG, (b) Fab, (c) F(ab)$_2$, (d) scFv, (e) diabody, (f) tribody, and (g) scFv–Fc. These formats vary in size, valency, and functional properties, influencing their half-life, tissue penetration, and effector functions.

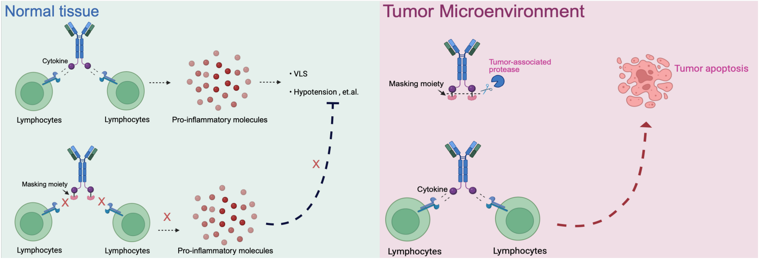

Figure S3. The schematic of prodrugs in normal tissue and tumor microenvironment. Normal tissue: Cytokines or immunocytokines without a masking moiety can activate lymphocytes systemically, leading to widespread release of pro-inflammatory mediators and dose-limiting toxicities. When masked, cytokine activity is blocked, preventing unwanted immune activation in healthy tissues. Tumor microenvironment: Protease-rich conditions allow cleavage of the masking moiety, restoring cytokine activity. The locally released cytokine activates tumor-infiltrating lymphocytes, driving antitumor immune responses and promoting tumor apoptosis.

REFERENCES

Christ, O., Seiter, S., Matzku, S., Burger, C., and Zöller, M. (2001). Efficacy of local versus systemic application of antibody-cytokine fusion proteins in tumor therapy. *Clinical cancer research* 7, 985–998
